# Rape Myth Acceptance and Its Link with Mental Health Problems in Older Victims of Sexual Violence

**DOI:** 10.3390/healthcare14193354

**Published:** 2026-10-08

**Authors:** Anne Nobels, Arne Van den Broeck, Elizaveta Fomenko, Ines Keygnaert

**Affiliations:** 1 Department of Psychiatry, Ghent University Hospital, 9000 Ghent, Belgium; arne.vandenbroeck@ugent.be; 2 Department of Geriatrics, Ghent University Hospital, 9000 Ghent, Belgium; 3 International Centre of Reproductive Health, Department of Public Health and Primary Care, Ghent University, 9000 Ghent, Belgium; elizaveta.fomenko@ugent.be (E.F.); ines.keygnaert@ugent.be (I.K.); 4 Centre of Expertise in Violence Research, Capacity Building and Care (VIORESC), Department of Public Health and Primary Care, Ghent University, 9000 Ghent, Belgium; 5 Women’s Clinic, Ghent University Hospital, 9000 Ghent, Belgium

**Keywords:** rape myths, rape acknowledgement, older persons, sexual assault, internalizing disorders, trauma

## Abstract

**Highlights:**

**What are the main findings?**

- Rape myths acceptance (RMA) was not independently associated with symptoms of depression, anxiety or PTSD in older victims of sexual violence (SV).
- Yet, higher RMA was linked with increased symptoms of depression and anxiety in victims who framed their SV experience as “wrong” or “a crime”, underscoring the importance of considering the interaction between RMA and subjective SV framing when assessing mental health in older victims.

**What is the implication of the main findings?**

- Practitioners should consider how and when older victims with high RMA are encouraged to frame their experiences as “wrong” or “a crime,” as premature reframing could potentially exacerbate symptoms of depression and anxiety. When such framing occurs, sensitively addressing rape myths may help mitigate mental health symptoms.

**Abstract:**

**Background/Objectives:** Rape myth acceptance (RMA) may influence how older victims perceive their experiences of sexual violence (SV) and, consequently, their mental health. This study examined the associations between RMA, the framing of SV experiences, and mental health outcomes among sexually victimised older adults, with the aim of informing clinical practice. **Methods:** We analysed data from 197 adults aged 70 years and older in Belgium who reported lifetime sexual victimisation. Information on sexual victimisation, mental health, the framing of SV experiences, and RMA was collected through structured face-to-face interviews. Sexual victimisation was assessed using behaviourally specific questions, while symptoms of depression, anxiety, and post-traumatic stress disorder (PTSD) were measured using validated instruments. **Results:** Higher levels of RMA were associated with more severe depressive symptoms (*p* = 0.031) and anxiety symptoms (*p* = 0.008) among participants who labeled their SV experience as “wrong” or “a crime.” In addition, RMA was linked to higher anxiety symptoms among participants reporting financial difficulties (*p* = 0.012). **Conclusions:** Our findings highlight the importance of considering how RMA interacts with subjective framing of SV when evaluating mental health outcomes in older victims. Practitioners should be cautious about encouraging older victims with high RMA to frame their experience as “wrong” or “a crime”, while actively challenging rape myths when such framing emerges. For those with high RMA who frame their experience as “just something that happened”, interventions should focus on reducing self-blame and preventing revictimization.

## 1. Introduction

Sexual violence (SV) represents a significant global public health concern [1]. Exposure to sexual victimisation across the life course, including in childhood and adulthood, is robustly associated with persistent adverse mental health outcomes [2], with evidence linking lifetime SV to depression, anxiety, and post-traumatic stress disorder (PTSD) in later life [3,4]. A Belgian study from 2021 reported that 8% of older adults experienced sexual victimisation in the past 12 months and 44% during their lifetime [5]. Given this high prevalence and substantial mental health burden, there is an urgent need to identify factors that modify the mental health consequences of SV in later life. Such evidence is essential to improve risk stratification and to inform interventions aimed at reducing its impact in older populations [6].

Research in student populations suggests that a victim’s rape myth acceptance (RMA) may moderate the mental health impact of sexual victimization [7,8,9]. Rape myths are widely held, but false stereotypical beliefs about rape [10]. They often concern the victim’s behaviour, appearance or character, the assailant’s intentions and the circumstances of the assault [11,12]. RMA reflects the extent to which individuals endorse these beliefs. Rape myths may serve cognitive and emotional functions, such as preserving self-esteem, reducing negative affect and rationalizing harmful behaviour [13]. Adult rape victims with high RMA are less likely to recognize their experience as aligning with their understanding of rape, even if it meets legal definitions of rape. They label the incidents as “miscommunication” or “bad sex”, leading to a phenomenon known as “unacknowledged rape” [13,14,15]. Unacknowledged victims are more vulnerable to revictimization [8] and are at greater risk of experiencing heightened self-blame, consequently suffering from increased shame, self-hatred and guilt [16], which are known risk factors for psychopathology [17].

Older adults may be especially prone to internalizing rape myths, as they grew up in an era of limited sexual health literacy where sexuality was taboo. Persistent taboos may foster deep-seated shame and self-blame, further hindering recognition of victimisation [18]. This dynamic has been shown to affect help-seeking behaviour and may increase vulnerability to adverse mental health outcomes, including depression, anxiety, PTSD, self-harm and suicide [19,20]. Moreover, society often perceives older adults as asexual, which contributes to the perception that they are less desirable and, by extension, at lower risk of SV compared to younger adults [21]. This sociocultural desexualisation renders older adults incompatible with both Christie’s notion of the ideal victim [22] and the dominant “real rape” stereotype. The latter typically imagines a rape victim as a young woman attacked at night by a stranger motivated by sexual gratification [6,21]. Consequently, for older adults, acknowledging their experiences as SV becomes even more difficult, further discouraging help-seeking and reporting to legal authorities [6,18].

It remains unclear whether rape myth acceptance (RMA) is associated with increased or decreased risk of mental health complaints following victimisation [7,8,9]. Evidence suggests that the impact of RMA may hinge on its interaction with rape acknowledgement [8], as sociocultural factors such as RMA shape both the experience of SV and the way it is remembered and conceptualised by victims [8,23]. This aligns with the information processing theory (IPT) of Resick and Schnicke [24], which posits that post-SV adjustment is shaped by both victims’ beliefs (e.g., rape myth acceptance) and the way they conceptualise (i.e., frame) the SV incident. Their theory posits that SV threatens the schematic beliefs people hold. This threat can be resolved by assimilating the event into one’s schemas (e.g., by minimizing the event as “bad sex”) or by accommodating (e.g., believing that “many men are good, but some are bad”) or overaccommodating (e.g., believing “all men are dangerous”) their schemas to the SV event [24]. Each of these cognitive adjustment strategies is associated with distinct mental health trajectories [25].

To date, research on the moderating role of RMA in mental health outcomes following SV has focused mainly on student samples [7,8,9], leaving its applicability to older adults uncertain. To our knowledge, no quantitative study has yet examined RMA in older adults, nor its association with mental health outcomes. Given the high prevalence and profound impact of SV in later life, the rapidly aging world population, and the limited understanding of factors moderating post-victimisation mental health outcomes in older adults, further research in this area is required. To address these gaps, this study examined the following research questions: (1) what is the association between rape myth acceptance (RMA) and mental health outcomes among sexually victimised older adults, and (2) does victims’ framing of their SV experiences moderate the relationship between RMA and mental health outcomes? As previous research suggests that acknowledgement and labelling influence how RMA affects mental health [8], we hypothesised that higher rape myth acceptance (RMA) would strengthen the association between SV and mental health outcomes, particularly among victims framing their experience as “wrong” or “a crime”. Our approach fills an important gap in the literature about mental health in sexually victimised older adults and allows us to formulate urgently needed evidence-based recommendations to strengthen professional capacity and improve care for older SV victims.

## 2. Materials and Methods

### 2.1. Participants

This study was conducted within the framework of a national research project on SV in the Belgian population aged 16 to 100 years. Between 8 July 2019 and 12 March 2020, structured face-to-face interviews were carried out with a representative sample of 513 older adults (≥70 years) across Belgium. Participants completed questions covering, among other domains, socio-demographic characteristics (Table 1), RMA, experiences of SV, and mental health outcomes. A comprehensive description of the development, validation, acceptability, and feasibility of the questionnaire in the Belgian population, including older adults, has been published previously [26].

Participants were recruited using a random-walk sampling procedure and were eligible if they resided in Belgium, were proficient in Dutch, French, or English, and demonstrated sufficient cognitive capacity to complete the interview [26,27]. Cognitive capacity was evaluated during the interview based on sustained attention and consistency of responses, including verification through a control item comparing reported age and year of birth. The sample included older adults from different living settings, including community dwellings, assisted living facilities, and nursing homes. All interviews were conducted in private by trained interviewers at participants’ place of residence. The overall participation rate was 34.0%. A detailed study protocol has been reported elsewhere [26].

Among the 513 participants, 227 (44.2%) described experiencing SV during their lifetime [5]. For this study, we specifically selected the older SV victims who provided complete responses to the measures on RMA, SV framing, and all sociodemographic variables. In total, 197 participants were included in the analyses.

### 2.2. Definitions and Measures

This study applied the World Health Organization (WHO) definition of SV, which differentiates between “hands-off” and “hands-on” forms and includes sexual harassment, sexual abuse involving physical contact without penetration, and (attempted) rape [1,28]. In light of emerging evidence on SV in older populations, this definition was extended to incorporate sexual neglect [29]. Sexual neglect is defined as the failure to ensure privacy, to respect a person’s sexual orientation or gender identity, to recognise older adults as sexual beings, or to provide opportunities for sexual expression [30]. Due to the lack of a standardized measure, sexual neglect was assessed through the item “touching in care”. Sexual victimisation was measured using behaviourally specific questions providing explicit descriptions of sexual acts to reduce ambiguity and enhance reliability [31] (see Appendix A). The SV items were adapted from existing surveys and fitted to the Belgian social and legal context [32,33,34,35]. Lifetime sexual victimisation was calculated by creating dichotomous variables for all 17 SV items, and was defined as exposure to any of these 17 items (yes/no) from birth until the time of the survey. We did not distinguish between hands-off and hands-on SV, due to a high level of multicollinearity between these two groups (variance inflation factor (VIF) >4).

Participants who reported lifetime SV were asked how they framed the SV experience that had the most impact on their lives as “just something that happened”, “wrong, but not a crime”, or “a crime” (see Appendix B). If participants reported a single incident, this was designated as the reference event. For the purpose of analysis, framing was dichotomised into “wrong or crime” versus “just something that happened.” Rape myth acceptance was measured using a short version of the Acceptance of Modern Myths about Sexual Aggression (AMMSA) scale [11]. Permission to use the AMMSA scale was obtained from the original authors. The scale was adapted with partially gender-neutral wording and reviewed for reliability and appropriateness by both an expert panel and older adults [26]. Participants rated 13 statements about SV on a 5-point Likert scale (1 = strongly disagree to 5 = strongly agree). RMA was dichotomized into “low” and “high” according to the sample’s mean AMMSA score [36].

Mental health outcomes were assessed using validated, internationally established scales that are freely available to the public. Depressive symptoms were measured with the Patient Health Questionnaire (PHQ-9) [37], while anxiety symptoms were assessed using the Generalised Anxiety Disorder scale (GAD-7) [38]; both refer to symptoms experienced in the two weeks prior to the interview. Post-traumatic stress disorder (PTSD) symptoms were evaluated using the Primary Care PTSD Screen for DSM-5 (PC-PTSD-5) [39], referring to the month preceding the interview. Participants were also asked about lifetime self-harm and lifetime suicide attempts (Appendix B).

### 2.3. Statistical Analysis

Statistical analyses were conducted using SPSS Statistics version 29. Descriptive statistics were computed for all study variables. Mental health outcomes were reported in categorical form, in line with their application in clinical practice.

Bivariate analyses were performed to compare the proportion of participants with “high RMA” and “wrong or crime” framing across the different variables of mental health. Chi-square tests were used, or Fisher’s exact tests when Chi-square assumptions were not met.

To examine the association between RMA and depression, anxiety, and PTSD, three separate linear regression analyses were performed, using PHQ-9, GAD-7, and PC-PTSD-5 scores as continuous outcome variables. In the first step, we conducted a linear regression analysis with all main effects. Suicide attempts and self-harm were not included as outcome measures because of their low prevalence, which would have resulted in limited statistical power. Multicollinearity was tested using the variance inflation factor, which indicated no violations.

In a second step, cross-product terms were constructed to explore potential moderating effects of sociodemographic characteristics (Table 1) and SV framing on the association between RMA and mental health outcomes. Cross-product terms were created between each moderator and RMA. Due to the limited sample size cross-product terms were added in a forward stepwise manner based on selection criteria (*p* < 0.05). The final model retained all main effects and only significant (*p* < 0.05) interaction effects.

## 3. Results

The study sample consisted of 197 sexually victimized older adults in Belgium. Their mean age was 78 years (SD = 6.0; range 70–95). The majority were female (74.1%), community-dwelling (91.9%), and had no or low formal education (68.0%). Financial difficulties were reported by 27.4%, illness or disability limiting daily activities by 45.2%, care dependency by 48.2% and low social support by 51.8% (Table 1).

**Table 1:** Sociodemographic characteristics of the study population (n = 197).

Variable		n (%) Study Population
Sex assigned at birth	Male	51 (25.9)
	Female	146 (74.1)
Age	70–79 y	120 (60.9)
	80–89 y	69 (35.0)
	90–99 y	8 (4.1)
Living situation	Community-dwelling	181 (91.9)
	Assisted living facility/nursing home	16 (8.1)
Country of birth	Belgium	182 (92.4)
	Other	15 (7.6)
Education level	No/low education ^a^	134 (68.0)
	Higher education	63 (32.0)
Financial status ^b^	Easy	143 (72.6)
	Difficult	54 (27.4)
Perceived health status	No disability/chronical illness	108 (54.8)
	Disability/chronical illness	89 (45.2)
Care dependency ^c^	No	102 (51.8)
	Yes	95 (48.2)
Social support ^d^	Low	102 (51.8)
	High	95 (48.2)

^a^ This group includes participants with no formal education or who completed primary education, secondary education, technical or vocational education, or religious schooling. ^b^ Assessed via self-reported ability to make ends meet. The four response categories (“with great difficulty,” “with some difficulty,” “fairly easily,” and “easily”) were dichotomised into “difficult” and “easy.” ^c^ Assessed by participants’ regular use of assistance for medical reasons. ^d^ Measured by number of confidants. The average number of confidants was 3. Low social support corresponds to 2 or less confidants, high social support to 3 or more confidants.

Regarding sexual victimisation, 48.2% experienced both hands-on and hands-off SV during their lifetime, 30.0% only hands-off SV and 21.8% only hands-on SV. Among the whole study population, 58.4% were victims of sexual abuse and 11.7% of (attempted) rape. Notably, 44.2% perceived the SV incident they identified as having had the most impact on their lives as “just something that happened”, while 55.8% considered it “wrong or a crime”. Framing responses by type of SV are reported in Appendix C. Regarding RMA, the mean score was 3.16 (SD = 0.4), with 48.7% of participants scoring above this mean (Table 2).

Among the participants, 21.3% reported mild, 7.6% moderate, and 3.6% (moderately) severe depressive symptoms. Anxiety symptoms were reported as mild by 19.8%, moderate by 6.6%, and (moderately) severe by 4.6%. In the month preceding the interview, 8.6% experienced three or more PTSD symptoms. Lifetime suicide attempts were reported by 5.6% and self-harm by 1.0%.

Participants with moderate and (moderately) severe anxiety symptoms were significantly more likely to frame their most impactful SV experience as ‘wrong’ or ‘a crime’ compared with those with lower anxiety levels. No significant results were found for other mental health outcomes. No significant differences in mental health outcomes were observed between participants with high versus low RMA. A detailed overview of mental health outcomes is presented in Table 3.

The linear regression analyses (Table 4) explored the associations between RMA and symptoms of depression, anxiety, and PTSD, with each outcome assessed separately. Depressive and PTSD symptoms were more pronounced among participants reporting financial difficulties or living with a chronic illness or disability, while anxiety symptoms were elevated specifically in those with chronic illness or disability. No significant associations were observed between mental health outcomes and sex assigned at birth, age, living situation, country of birth, educational level, care dependency, or social support.

RMA was not independently associated with depression, anxiety or PTSD. Among participants who framed their SV experience as “wrong or a crime”, higher RMA was associated with increased symptoms of depression and anxiety. Additionally, higher RMA was linked to elevated anxiety symptoms in participants reporting financial difficulties.

## 4. Discussion

### 4.1. Discussion of Main Results

Based on a sample of 197 sexually victimised older adults in Belgium, we examined the association between RMA and depression, anxiety, PTSD, suicide attempts, and self-harm. We further assessed the moderating role of SV framing and sociodemographic characteristics in this association, recognizing these as crucial factors in understanding mental health outcomes in older SV victims.

Bivariate analyses revealed that participants experiencing moderate to severe anxiety were more inclined to interpret their most impactful SV experience as ‘wrong’ or ‘a crime’ than those with lower anxiety levels. As these findings are based on a single study and existing evidence on the relationship between acknowledgement and mental health remains inconclusive [40], they should be viewed as preliminary. Nevertheless, our study is among the first to investigate framing in older adults, thereby expanding the limited literature on this topic and contributing to future research into the role of framing in mental health symptomatology.

Our regression analyses corroborate previous findings indicating that financial hardship is associated with an increased risk of depression and PTSD in older adults [41,42]. Regarding anxiety, our results show that higher RMA is linked to greater anxiety among SV victims experiencing economic strain. We did not find literature on this direct interaction, however, prior research indicates that lower income is associated with higher RMA [43] and that RMA reflects adherence to traditional gender roles in both women and men [13,43]. Research has shown that older adult women and men who adhere more strongly to a ‘traditional gender role’, tend to use more passive and maladaptive coping strategies when confronted with adversities, which in turn leads to more mood disorders [44]. It is therefore plausible that this adherence to ‘traditional gender roles’, associated with high RMA, undermines effective coping strategies and exacerbates anxiety due to financial hardship [43,44]. Further research is needed to examine this hypothesis. No significant associations were observed between education level, RMA, or mental health outcomes, nor were any interaction effects significant. Given the documented links between education level, RMA, and mental health [45,46], this finding is noteworthy and may be attributable to the predominance of participants with no or low education in our sample.

Furthermore, our regression analyses showed that RMA was not independently associated with depression, anxiety or PTSD, but that higher RMA was associated with increased symptoms of depression and anxiety among participants who framed their SV experience as “wrong or a crime”. This result is consistent with the IPT of Resick and Schnicke [24] and highlights the importance of this dynamic for the moderation of mental health symptomatology. As stronger endorsement of rape myths has been linked to greater victim-blaming attitudes [13], individuals with high RMA may internalize self-blame for the perceived wrongdoing or criminality of the event. This self-blame may contribute to heightened psychological distress [17]. In victims who framed the SV event as ‘just something that happened’, self-blame may be absent despite high RMA, as ‘there is nothing to be accountable for’. Among victims with low RMA in our sample, framing had no effect on mental health outcomes. This suggests that when rape myths are not endorsed, the way the SV incident is conceptualized may be irrelevant. This may be a consequence of a reduced tendency toward victim blaming and, consequently, self-blame, in victims with low RMA [13].

Our results align with previous research that found no direct association between RMA and mental health problems [7,8], but also reported that higher RMA (subscale) scores were linked to increased depressive symptoms in acknowledged victims, without observing such effect in unacknowledged victims [8]. Although the nuanced difference between framing and acknowledgement, Wilson et al. [8] share our assumption that these results reflect variations in self-blame related with higher RMA. Valdespino et al. [9] reported paradoxal results compared to ours and previous research [7,8] by showing higher depressive- and PTSD-symptoms in student victims of SV with low RMA (subscale) scores. Differences with our results may be explained by differences in population, considering a part of their study population had no history of SV. Furthermore, they didn’t include framing as a moderating factor, which appears to be essential in the moderating effect of RMA [9].

### 4.2. Limitations

This study has some limitations. The small sample size limits statistical power, warranting cautious interpretation of the findings. Due to this limited statistical power, we were unable to examine whether the observed associations differed by sex assigned at birth. Therefore, future research with larger samples, particularly including older male victims, is warranted to investigate potential gender differences in the associations between rape myth acceptance (RMA), the framing of SV, and mental health outcomes. In addition, the dichotomization of the independent variables in the regression analysis reduces the statistical precision of our results.

Since our approach was exploratory, a forward stepwise regression model was used, which has an intrinsic risk of overfitting. Therefore, our results should be interpreted with caution and validated in future studies using similar and different samples.

Concerning study-design, our study’s cross-sectional design precludes causal inferences between sexual victimisation, mental health outcomes and RMA in later life. Framing and mental health problems may influence each other bidirectionally. For example, individuals with higher levels of anxiety may reinterpret experiences differently over time. Therefore, our results should be interpreted in light of these limitations.

SV was reported retrospectively and we didn’t differentiate between SV that happened during childhood, adulthood or old age. This distinction may be relevant since symptomatology of mental health problems [47]; RMA-development [48] and labelling of SV [40] can all be influenced by the timing of SV and evolve during lifetime. Additionally, religion and relationship status were not included in the regression analysis, and we did not differentiate based on the relationship between victim and assailant. This relation has been shown to influence labelling [49] and may influence the alignment between the SV and rape scripts [14]. Furthermore, due to substantial collinearity between hands-off and hands-on SV (VIF > 4), we were unable to distinguish between different types of SV in our analyses. Although similar framing patterns emerged across SV forms in our data (Appendix C), prior research suggests that framing may differ across SV types, depending on how closely they fit the “real rape” script [14]. The lack of differentiation in our analyses may therefore have influenced our findings. Future studies should consider distinguishing between SV types and examining how these relate to RMA and framing. Furthermore, generalisability is limited given the culturally embedded nature of RMA. Whereas most previous studies on rape myths have used the Illinois Rape Myth Acceptance Scale (IRMAS) [7,8,13,50], we used the AMMSA scale, as it was found to be more acceptable to our study population [26] and is better aligned with the European and Belgian context [11,14]. Consequently, the use of a different instrument limits the direct comparability of our findings with those of previous studies using the IRMAS. This should be taken into account when interpreting and comparing our results with the existing literature. Future cross-cultural research using comparable methodologies, measurement instruments, and definitions of SV is encouraged to further examine these issues [51].

### 4.3. Recommendations for Clinical Practice

Our findings generate clinical implications for practice. First, a supportive, client-centred exploration of levels of rape myth acceptance (RMA) among older SV victims by practitioners appears important. For individuals with high RMA who do not perceive their SV experiences as “wrong” or a “crime,” immediate reframing of these experiences may not always be appropriate, as this could potentially exacerbate symptoms of depression and anxiety. Conversely, when such framing occurs, practitioners may sensitively challenge rape myths as part of interventions that aim to reduce depressive and anxiety symptoms. For victims with high RMA who do not acknowledge their experience as “wrong” or a “crime,” interventions might focus on reducing self-blame and supporting coping strategies, as well as on the prevention of revictimisation.

As our results align with the IPT, Cognitive Processing Therapy (CPT) emerges as a potential treatment, as this is an IPT-grounded form of cognitive behavioural therapy (CBT) with evidence-based efficacy for adult SV victims [52]. By targeting maladaptive cognitions that sustain distress, CPT directly reflects the mechanisms demonstrated in our results [52]. Given CBT, and so presumably CPT, has proven to be beneficial in older adults [53] and that interventions to reduce RMA have shown effectiveness [54] we offer therapeutic tools to approach older SV victims. In this way, our study contributes to the urgently needed capacity building for healthcare professionals supporting older victims of SV.

## 5. Conclusions

In older SV victims, higher RMA is associated with more severe symptoms of depression and anxiety, particularly in those framing their experience as “wrong” or “a crime.” These findings emphasize the importance of considering how RMA interacts with victims’ subjective framing of SV when assessing mental health outcomes in later life. From a clinical perspective, our findings suggest that practitioners may wish to carefully consider how and when older victims with high RMA are encouraged to frame their experiences as “wrong” or “a crime,” as premature reframing could potentially exacerbate symptoms of depression and anxiety. Conversely, when such framing occurs, sensitively addressing rape myths may help mitigate mental health symptoms. For individuals with high RMA who do not acknowledge their experience as SV, interventions may instead benefit from focusing on reducing self-blame and preventing revictimisation.

By offering these practical recommendations, our study contributes to the urgent need for capacity building of healthcare professionals working with older SV victims. Ultimately, these insights not only advance academic understanding but also support more tailored and effective care for this vulnerable population.

## Figures and Tables

**Table 2:** Sexual victimisation and RMA (n = 197).

Variable		n (%) Study Population
Sexual victimization	Both hands-off and hands-on	95 (48.2)
	Only hands-off	59 (30.0)
	Only Hands-on	43 (21.8)
	*Sexual abuse*	115 (58.4)
	(*Attempted*) *rape*	23 (11.7)
Framing SV	Just something that happened	87 (44.2)
	Wrong or a crime	110 (55.8)
RMA ^a^	Low	101 (51.3)
	High	96 (48.7)

^a^ Measured using the Acceptance of Modern Myths about Sexual Aggression (AMMSA) scale. Dichotomised based on the mean (min-max): 3.16 (1.77–4.31).

**Table 3:** Association of Framing and Rape myth acceptance with depression, anxiety and PTSD (n = 197).

Item	Scale (α)	Outcome	n (%)	Framing Wrong/Crime ^a^ n (%)	*p*-Value	High RMA ^b^ n (%)	*p*-Value
Depression ^c^	PHQ-9	No	133 (67.5)	70 (52.6)	0.615 °	60 (45.1)	0.400 °
	(0.755)	Mild	42 (21.3)	26 (61.9)		25 (59.5)	
		Moderate	15 (7.6)	9 (60.0)		7 (46.7)	
		Moderately severe/severe	7 (3.6)	5 (71.4)		4 (57.1)	
Anxiety ^d^	GAD-7	No	136 (69.0)	70 (51.5)	**<0.001 °**	62 (45.6)	0.262 °
	(0.835)	Mild	39 (19.8)	19 (48.7)		21 (53.9)	
		Moderate	13 (6.6)	13 (100.0) *		6 (46.2)	
		Moderately severe/severe	9 (4.6)	8 (88.9) *		7 (77.8)	
PTSD ^e^	PC-PTSD-5	No	180 (91.4)	100 (55.6)	0.806	87 (48.3)	0.802
	(0.600)	Yes	17 (8.6)	10 (58.8)		9 (52.9)	
Suicide attempts		No	186 (94.4)	103 (55.4)	0.758 °	89 (57.9)	0.364 °
lifetime		Yes	11 (5.6)	7 (63.6)		7 (63.6)	
Self-harm		No	195 (99,0)	109 (55.9)	1.000 °	95(48.7)	1.000 °
lifetime		Yes	2 (1.0)	1 (50.0)		1 (50.0)	

^a^ The proportion of participants who framed their most impactful experience of SV as wrong or a crime within the different categories of depression, anxiety, PTSD, lifetime suicide attempts and lifetime self-harm; ^b^ The proportion of participants that scored above average (AMMSA > 3.16) within the different categories of depression, anxiety, PTSD, lifetime suicide attempts and lifetime self-harm; ^c^ PHQ-9: No (0–4), Mild (5–9), Moderate (10–14), Moderately severe—Severe (≥15); ^d^ GAD-7: No (0–4), Mild (5–9), Moderate (10–14), Severe (≥15); ^e^ PC-PTSD-5: No (0–2), Yes (3–5); * Significant difference was found in this subcategory, statistically significant results are indicated in bold; ° Fisher’s Exact Test given the conditions for chi-square are not met: *p*-value. Note: Because the comparisons in this table involved 2 sets of 5 independent tests, we adopted a Bonferroni-corrected significance level of 0.050/5 = 0.010 for these analyses. Abbreviations: RMA = Rape Myth Acceptance; PTSD = Post-traumatic Stress Disorder; α = Cronbach’s Alfa; PHQ-9 = Patient Health Questionnaire-9; GAD-7 = General Anxiety Disorder-7; PC-PTSD-5 = Primary Care PTSD Screen for DSM-5.

**Table 4:** Association of Rape myth acceptance and framing with depression, anxiety and PTSD (n = 197).

	Depression (PHQ-9)			Anxiety (GAD-7)			PTSD (PC-PTSD-5)		
	Coeff.	95% CI	*p*-Value	Coeff.	95% CI	*p*-Value	Coeff.	95% CI	*p*-Value
**Main effects**									
RMA	−1.466	−3.122, 0.190	0.082	−0.179	−3.475, 0.255	0.090	−0.132	−0.425, 0.162	0.377
Framing	−0.629	−2.179, 0.921	0.425	0.059	−1.063, 2.134	0.509	0.236	−0.056, 0.528	0.113
Financial status	1.613	0.368, 2.858	**0.011**	−0.229	−2.143, 1.698	0.819	0.426	0.098, 0.754	**0.011**
Perceived health status	2.817	1.707, 3.927	**<0.001**	0.288	1.464, 3.746	**<0.001**	0.456	0.165, 0.748	**0.002**
**Cross-products terms & interaction effect**									
RMA * Framing	3.265	0.892, 5.638	**0.007**	2.657	0.369, 4.945	**0.023**	−	−	−
*Something that happened*	−1.398	−3.022, 0.226	0.091	−0.410	−1.714, 0.895	0.534	−	−	−
*Wrong/crime*	1.866	0.177, 3.556	**0.031**	2.639	0.696, 4.582	**0.008**	−	−	−
RMA * Financial status	-	-	-	3.767	−0.983, 6.550	**0.008**	-	-	-
*Easy*	-	-	-	0.088	−1.213, 1.390	0.893	-	-	-
*Difficult*	-	-	-	3.855	0.869, 6.841	**0.012**	-	-	-

Note: In this table we describe the final forward linear regression models for depression anxiety and PTSD, in which all main and significant (*p* < 0.05) interaction effects were retained. The coefficient describes the variability of the outcome variable in comparison to a reference value. This reference value is for RMA “AMMSA < 3.16”, for Framing “framed as just something that happened”, for Perceived health status “no chronic illness/disability”, for Financial status “easy”. The bold values are statistically significant. Abbreviations: CI = Confidence Interval; Coeff = Coefficient; RMA = Rape myth acceptance; GAD-7 = General Anxiety Disorder-7; PC-PTSD-5 = Primary Care PTSD Screen for DSM-5; PHQ-9 = Patient Health Questionnaire-9; PTSD = Posttraumatic Stress Disorder.

## Data Availability

The data presented in this study are available from the corresponding author upon reasonable request due to privacy and confidentiality concerns, particularly given the sensitive nature of the personal data collected, including information on mental health and experiences of sexual violence. Additionally, at the time of the interviews, the informed consent form did not include consent for data sharing beyond the research team.

## Abbreviations

The following abbreviations are used in this manuscript:

RMA	Rape Myths Acceptance
SV	Sexual Violence
PTSD	Post-traumatic Stress Disorder
IPT	Information Processing Theory
VIF	Variance Inflation Factor
AMMSA	Acceptance of Modern Myths about Sexual Aggression
PHQ-9	Patient Health Questionnaire
GAD-7	General Anxiety Disorder scale
PC-PTSD-5	Primary Care PTSD Screen for DSM-5
SD	Standard Deviation
CPT	Cognitive Processing Therapy
CBT	Cognitive Behavioural Therapy

## Appendix A. Detailed Outcome Measurements Sexual Victimization Reprinted from Ref. [5]

Hands-off sexual victimisation (no physical contact):

- Sexual staring: Someone stared at me in a sexual way or looked at my intimate body parts (e.g., breasts, vagina, penis, anus) when I didn’t want it to happen.
- Sexual innuendo: Someone made teasing comments of a sexual nature about my body or appearance even though I didn’t want it to happen.
- Showing sexual images: Someone showed me sexual or obscene materials such as pictures, videos, directly or over the internet (including email, social networks and chat platforms) even though I didn’t want to look at them. This does not include mass mailings or spam.
- Sexual calls or texts: Someone made unwelcome sexual or obscene phone calls or texts to me.
- Voyeurism: I caught someone watching me, taking photos or filming me when I didn’t want it to happen while I was undressing, nude or having sex.
- Distribution of sexual images: Someone distributed naked pictures or videos of me directly or over the internet (including email, social networks and chat platforms) when I didn’t want it to happen.
- Exhibitionism: Someone showed their intimate body parts (e.g., breasts, vagina, penis, anus) to me in a sexual way and/or masturbated in front of me when I didn’t want to see it.
- Forcing to show intimate body parts: Someone made me show my intimate body parts (e.g., breasts, vagina, penis, anus) online or face-to-face when I didn’t want to do it.

Hands-on sexual victimization:

Sexual abuse (physical contact but no penetration).

- Kissing: Someone kissed me against my will.
- Touching in care: Someone touched my intimate body parts (e.g., breasts, vagina, penis, anus) during care against my will.
- Fondling/rubbing: Someone fondled or rubbed up against my intimate body parts (e.g., breasts, vagina, penis, anus) against my will.
- Forced undressing: Someone removed (some of) my clothes against my will

Rape and attempted rape (physical contact with attempted or completed penetration):

- Oral penetration: Someone had oral sex with me or made me give oral sex against my will.
- Attempt of oral penetration: Someone tried, but did not succeed, to have oral sex with me or tried to make me give oral sex against my will.
- Vaginal or anal penetration: Someone put their penis, finger(s) or object(s) into my vagina or anus against my will.
- Attempt of vaginal or anal penetration: Someone tried, but did not succeed to put their penis, finger(s) or object(s) into my vagina or anus against my will.
- Forcing to penetrate: Someone made me put my penis, finger(s) or object(s) into their (or someone’s) vagina or anus against my will.

## Appendix B. Questions on Framing of Sexual Violence, Suicide Attempts and Self-Harm Adapted from Refs. [5,42]

Question on framing of sexual violence:

Do you consider this to be…

- A crime, a punishable offence
- Wrong but not a crime
- Just something that happened

Questions on suicide attempts and self-harm:

- Have you ever made an attempt to take your life or kill yourself?
- Deliberately harmed yourself in any way but not with the intention of killing yourself?

## Appendix C. Framing per Form of Sexual Violence

	**Just Something That Happened** **n (% of Total Victims of** **This Type of SV)**	**Crime/Wrong** **n (% of Total Victims of** **This Type of SV)**
Any hands-off (n = 124)	49 (39.52)	75 (60.48)
Any hands-on (n = 138)	59 (42.75)	79 (57.25)

## References

[1] Krug E.G., Mercy J.A., Dahlberg L.L., Zwi A.B. (2002). The world report on violence and health. Lancet.

[2] Oram S., Khalifeh H., Howard L.M. (2017). Violence against women and mental health. Lancet Psychiatry.

[3] Ege M.A., Messias E., Thapa P.B., Krain L.P. (2015). Adverse childhood experiences and geriatric depression: Results from the 2010 BRFSS. Am. J. Geriatr. Psychiatry.

[4] Rapsey C.M., Scott K.M., Patterson T. (2019). Childhood sexual abuse, poly-victimization and internalizing disorders across adulthood and older age: Findings from a 25-year longitudinal study. J. Affect Disord..

[5] Nobels A., Cismaru-Inescu A., Nisen L., Hahaut B., Beaulieu M., Lemmens G., Adam S., Schapansky E., Vandeviver C., Keygnaert I. (2021). Sexual violence in older adults: A Belgian prevalence study. BMC Geriatr..

[6] Bows H., Westmarland N. (2017). Rape of older people in the United Kingdom: Challenging the ‘real-rape’stereotype. Br. J. Criminol..

[7] Dworkin E.R., Sessarego S.N., Pittenger S.L., Edwards K.M., Banyard V.L. (2017). Rape Myth Acceptance in Sexually Assaulted Adolescents’ School Contexts: Associations with Depressed Mood and Alcohol Use. Am. J. Community Psychol..

[8] Wilson L.C., Newins A.R., White S.W. (2018). The impact of rape acknowledgment on survivor outcomes: The moderating effects of rape myth acceptance. J. Clin. Psychol..

[9] Valdespino-Hayden Z., Walsh K., Lowe S.R. (2022). Rape Myth Acceptance Buffers the Association Between Sexual Assault and Posttraumatic Stress Disorder Symptoms Among College Students. J. Interpers. Violence.

[10] Burt M.R. (1980). Cultural myths and supports for rape. J. Pers. Soc. Psychol..

[11] Gerger H., Kley H., Bohner G., Siebler F. (2007). The acceptance of modern myths about sexual aggression scale: Development and validation in German and English. Aggress. Behav..

[12] Lonsway K.A., Fitzgerald L.F. (1994). Rape myths: In review. Psychol. Women Q..

[13] Peterson Z.D., Muehlenhard C.L. (2004). Was it rape? The function of women’s rape myth acceptance and definitions of sex in labeling their own experiences. Sex. Roles.

[14] LeMaire K.L., Oswald D.L., Russell B.L. (2016). Labeling sexual victimization experiences: The role of sexism, rape myth acceptance, and tolerance for sexual harassment. Violence Vict..

[15] Frese B., Moya M., Megías J.L. (2004). Social perception of rape: How rape myth acceptance modulates the influence of situational factors. J. Interpers. Violence.

[16] Arata C.M., Burkhart B.R. (1996). Post-Traumatic Stress Disorder Among College Student Victims of Acquaintance Assault. J. Psychol. Hum. Sex..

[17] Kubany E.S., Manke F.P. (1995). Cognitive therapy for trauma-related guilt: Conceptual bases and treatment outlines. Cogn. Behav. Pract..

[18] Nobels A., De Schrijver L., Van Landuyt M., Vandeviver C., Lemmens G.M.D., Beaulieu M., Keygnaert I. (2024). In the End You Keep Silent": Help-Seeking Behavior Upon Sexual Victimization in Older Adults. J. Interpers. Violence.

[19] Callow T.J., Moffitt R.L., Neumann D.L. (2021). External shame and its association with depression and anxiety: The moderating role of self-compassion. Aust. Psychol..

[20] Cunningham K.C. (2020). Shame and guilt in PTSD. Emotion in Posttraumatic Stress Disorder.

[21] (2021). Przybylo E: Ageing asexually: Exploring desexualisation and ageing intimacies. Sex and Diversity in Later Life.

[22] (1986). Christie N: The ideal victim. From Crime Policy to Victim Policy: Reorienting the Justice System.

[23] (1987). Estrich S: Real Rape.

[24] Resick P.A., Schnicke M.K. (1992). Cognitive processing therapy for sexual assault victims. J. Consult Clin. Psychol..

[25] (2007). Littleton H: An evaluation of the coping patterns of rape victims: Integration with a schema-based information-processing model. Violence Against Women.

[26] Nobels A., Cismaru-Inescu A., Nisen L., Hahaut B., Lemmens G.M., Vandeviver C., Keygnaert I. (2022). Challenges in conducting sexual health and violence research in older adults beyond the General Data Protection Regulation: A Belgian case study. J. Interpers. Violence.

[27] Lewis-Beck M., Bryman A.E., Liao T.F. (2003). The Sage Encyclopedia of Social Science Research Methods.

[28] World Health Organization (2021). Violence Against Women Prevalence Estimates, 2018: Global, Regional and National Prevalence Estimates for Intimate Partner Violence Against Women and Global and Regional Prevalence Estimates for Non-partner Sexual Violence Against Women.

[29] Nobels A., Vandeviver C., Beaulieu M., Inescu A.C., Nisen L., Van Den Noortgate N., Vander Beken T., Lemmens G., Keygnaert I. (2020). “Too grey to be true?” Sexual violence in older adults: A critical interpretive synthesis of evidence. Int. J. Environ. Res. Public Health.

[30] Montreal C.W., Helpline E.M. Research Chair on Mistreatment of Older Adults; Ministère de la Famile Saa, Gouver Nement du Québec. Leading Practice to Counter the Mistreatment of Older Adults 2017. https://www.inspq.qc.ca/en/mistreatment-older-adults.

[31] Depraetere J., Vandeviver C., Beken T.V., Keygnaert I. (2020). Big boys don’t cry: A critical interpretive synthesis of male sexual victimization. Trauma Violence Abus..

[32] Krahé B., Berger A., Vanwesenbeeck I., Bianchi G., Chliaoutakis J., Fernández-Fuertes A.A., Fuertes A., de Matos M.G., Hadjigeorgiou E., Haller B. (2015). Prevalence and correlates of young people’s sexual aggression perpetration and victimisation in 10 European countries: A multi-level analysis. Cult. Health Sex..

[33] Koss M.P., Gidycz C.A. (1985). Sexual experiences survey: Reliability and validity. J. Consult. Clin. Psychol..

[34] Keygnaert I., Dias S.F., Degomme O., Devillé W., Kennedy P., Kováts A., De Meyer S., Vettenburg N., Roelens K., Temmerman M. (2015). Sexual and gender-based violence in the European asylum and reception sector: A perpetuum mobile?. Eur. J. Public Health.

[35] Depraetere J., Inescu A.C., De Schrijver L., Nobels A., Keygnaert I., Vandeviver C. (2020). Measuring Sexual Victimization and Perpetration in Today’s Society: Modifications to the Sexual Experiences Survey. https://osf.io/preprints/socarxiv/cgevu_v1.

[36] Aiken L.S. (1991). Multiple Regression: Testing and Interpreting Interactions.

[37] Kroenke K., Spitzer R.L., Williams J.B. (2001). The PHQ-9: Validity of a brief depression severity measure. J. General. Intern. Med..

[38] Löwe B., Decker O., Müller S., Brähler E., Schellberg D., Herzog W., Herzberg P.Y. (2008). Validation and standardization of the Generalized Anxiety Disorder Screener (GAD-7) in the general population. Med. Care.

[39] Prins A., Bovin M.J., Smolenski D.J., Marx B.P., Kimerling R., Jenkins-Guarnieri M.A., Kaloupek D.G., Schnurr P.P., Kaiser A.P., Leyva Y.E. (2016). The primary care PTSD screen for DSM-5 (PC-PTSD-5): Development and evaluation within a veteran primary care sample. J. General. Intern. Med..

[40] Wilson L.C., Miller K.E. (2016). Meta-analysis of the prevalence of unacknowledged rape. Trauma Violence Abus..

[41] Cole M.G., Dendukuri N. (2003). Risk factors for depression among elderly community subjects: A systematic review and meta-analysis. Am. J. Psychiatry.

[42] Nobels A., Lemmens G., Thibau L., Beaulieu M., Vandeviver C., Keygnaert I. (2022). “Time Does Not Heal All Wounds”: Sexual Victimisation Is Associated with Depression, Anxiety, and PTSD in Old Age. Int. J. Environ. Res. Public Health.

[43] Sahin D., Güner Ö. (2023). Determinants of society’s rape myths acceptance. OPUS J. Soc. Res..

[44] Vafaei A., Ahmed T., Freire A., Zunzunegui M.V., Guerra R.O. (2016). Depression, sex and gender roles in older adult populations: The International Mobility in Aging Study (IMIAS). PLoS ONE.

[45] Süssenbach P., Bohner G. (2011). Acceptance of sexual aggression myths in a representative sample of German residents. Aggress. Behav..

[46] Prina F., Schatz-Stevens J.N. (2020). Sexism and Rape Myth Acceptance: The Impact of Culture, Education, and Religiosity. Psychol. Rep..

[47] Dunn E.C., Nishimi K., Powers A., Bradley B. (2017). Is developmental timing of trauma exposure associated with depressive and post-traumatic stress disorder symptoms in adulthood?. J. Psychiatr. Res..

[48] Cooke E.M., Lewis R.H., Hayes B.E., Bouffard L.A., Boisvert D.L., Wells J., Kavish N., Woeckener M., Armstrong T.A. (2022). Examining the Relationship Between Victimization, Psychopathy, and the Acceptance of Rape Myths. J. Interpers. Violence.

[49] Kahn A.S., Jackson J., Kully C., Badger K., Halvorsen J. (2003). Calling it rape: Differences in experiences of women who do or do not label their sexual assault as rape. Psychol. Women Q..

[50] Payne D.L., Lonsway K.A., Fitzgerald L.F. (1999). Rape myth acceptance: Exploration of its structure and its measurement using the Illinois Rape Myth Acceptance Scale. J. Res. Personal..

[51] Suarez E., Gadalla T.M. (2010). Stop blaming the victim: A meta-analysis on rape myths. J. Interpers. Violence.

[52] Resick P.A., LoSavio S.T., Monson C.M., Kaysen D.L., Wachen J.S., Galovski T.E., Stirman S.W., Nixon R.D., Chard K.M. (2024). State of the science of cognitive processing therapy. Behav. Ther..

[53] Jayasekara R., Procter N., Harrison J., Skelton K., Hampel S., Draper R., Deuter K. (2015). Cognitive behavioural therapy for older adults with depression: A review. J. Ment. Health.

[54] Hudspith L.F., Wager N., Willmott D., Gallagher B. (2023). Forty years of rape myth acceptance interventions: A systematic review of what works in naturalistic institutional settings and how this can be applied to educational guidance for jurors. Trauma Violence Abus..

